# Design of tractor virtual test system based on high-level architecture technology

**DOI:** 10.1371/journal.pone.0293229

**Published:** 2023-10-23

**Authors:** Xianghai Yan, Chengyan Shang, Junjiang Zhang, Liyou Xu

**Affiliations:** 1 College of Vehicle and Traffic Engineering, Henan University of Science and Technology, Luoyang, China; 2 State Key Laboratory of Intelligent Agricultural Power Equipment, Luoyang, China; University of Namibia, NAMIBIA

## Abstract

The limitations of the tractor virtual test system are evident in various aspects, including model reuse, system expansion, offsite interconnection, and virtual reality verification. To address these challenges, a distributed virtual test system for tractors based on the high-level architecture (HLA) is proposed. Involve analyzing the hardware structure and the tractor virtual test system, constructing the system federation and its members, and designing the federated object model (FOM) and simulation object model (SOM) tables. The system integrates multi-domain commercial software and enables real-time virtual testing through TCP/IP interconnection of multiple machines. To evaluate the system’s performance, a virtual test of the tractor’s reversing clutch engagement performance is conducted. The system’s simulation performance and data transmission delay are thoroughly tested and analyzed. The results indicate that when the system’s data volume reaches 5000KB, the data delay is 9.7ms, which satisfies the requirement of not exceeding 10ms for tractor virtual testing delay. The virtual test of the reversing clutch power reversal process demonstrates that it lasts 0.7s, with the vehicle speed changing from -3.5km/h to 3.5km/h, the forward gear piston oil pressure increasing from 0MPa to 5MPa, and the peak impact degree reaching 17m/s^3^. The slip work during the reversing process is measured to be 21kJ. Furthermore, the gray correlation method is employed to compare the virtual test results with the bench test results, confirming their consistency. The power reversal process exhibits relatively smooth speed changes overall. Therefore, the tractor power shift transmission (PST) reversing clutch virtual test model operates effectively within the HLA-based tractor virtual test system.

## Introduction

Test verification technology plays a crucial role in the entire lifecycle of tractor product development. It is essential in the development of tractor products as it allows for the assessment of their performance and guides the optimization of tractor design [1]. However, traditional tractor test technology poses challenges such as high costs, energy consumption, and demanding test equipment requirements, resulting in low efficiency in tractor product development [2, 3]. To address these issues, the application of computer technology and virtual test technology in new tractor product development has emerged as an advanced approach. Technologies like virtual reality (VR), virtual prototyping (VP), and virtual instrument (VI) are currently applied in the tractor virtual test process [4–6]. Nevertheless, the tractor virtual test involves multiple fields. Due to varying modeling principles and data transfer protocols used among commercial software in each field, different systems’ commercial software can only communicate through the application program interface (API) by exchanging files for joint simulation. This limits the real-time communication with the test equipment during the tractor virtual test [7]. Consequently, the tractor virtual test faces restrictions in model reuse, system expansion, remote interconnection, and combining virtual reality verification, ultimately impeding its efficiency. To overcome these limitations, it is imperative to establish integrated tractor test equipment and existing models within a comprehensive tractor virtual test system. Such an approach would significantly enhance the efficiency of tractor virtual testing and reduce the new product development cycle for tractors, carrying practical significance.

High-level architecture (HLA) is a widely recognized technology for modeling and simulation in the defense field, initially proposed by the Defense Modeling and Simulation Office of the U.S. Department of Defense in October 1995 [8]. HLA serves as a simulation framework that encompasses diverse simulation systems across various domains, allowing for enhanced interoperability, as well as the reusability of models between different simulation systems. Additionally, HLA can adapt to emerging technologies to address the simulation requirements of complex systems. Since its inception, HLA has garnered significant attention from both domestic and international researchers. Hu et al. extended the operational support environment by incorporating the Simulink model as a federate, resulting in the development of a distributed simulation system for rockets based on HLA [9]. Yuan et al. proposed a distributed perception technology grounded in HLA for an augmented reality (AR) soldier training system and carried out practical verification [10]. Wu et al. presented a design methodology for a hybrid simulation platform that integrates information physics and the power system, with a specific focus on the tightly coupled relationship between the power system and information network in a ship’s integrated power system. They successfully achieved information physics hybrid simulation using HLA as the foundation [11]. Shi et al. proposed a mission simulation platform for manned spacecraft based on HLA. The platform encompasses essential functionalities such as operation management and flight command, along with multidisciplinary simulation models covering orbit dynamics and other aspects [12]. Gu et al. established a multi-radar network information fusion simulation system using HLA, conducting system testing that analyzed the application of HLA and multi-radar network detection for stealth targets [13]. Furthermore, Wang et al. researched airborne laser weapons. Leveraging the characteristics of such weapons and the principles of HLA, they designed a simulation system that comprises ballistic missiles, carrier aircraft, and airborne Laser weapons [14]. Overall, these studies exemplify the extensive utilization of HLA in various simulation contexts, demonstrating its effectiveness and versatility as a technology for modeling and simulation in the defense field [15].

By the requirements of tractor testing, the generic tractor virtual test system must encompass various functions such as distributed modeling, a test operation support framework, data management, test process management, and test result evaluation. Additionally, it should possess attributes of real-time capability, reusability, interoperability, and expandability [16]. By employing HLA technology, the tractor virtual test system standardizes the interaction process among distinct commercial software simulation models. This enables different domain simulation models to exchange information based on a unified standard, thereby enhancing the reusability and interoperability of the tractor virtual test system.

This paper focuses on the architecture analysis of the tractor virtual test system and emphasizes the federation design approach. The aim is to address the limitation of traditional virtual test technology in conducting real-time electromechanical-hydraulic joint simulations. To accomplish this, an HLA-based tractor virtual test system is established. The system’s performance is evaluated using the tractor PST reversing clutch test as a case study.

## Analysis of tractor virtual test system based on HLA

### Main functions and features of the system based on HLA

The HLA-based tractor virtual test system incorporates the mechanical, hydraulic, and control modules of the tractor systems as fundamental virtual test units. Each virtual test unit, in conjunction with the virtual test system auxiliary module, constitutes a comprehensive function within the tractor virtual test system. These virtual test units operate within distinct virtual test environments to carry out diverse tests related to tractor performance. The system employs a test results evaluation function to assess the effectiveness of the virtual tests conducted. Additionally, it serves as a simulation test platform for the development of new tractor products and components. Utilizing multi-domain real-time simulation enables the evaluation of performance for new tractor products. This system also guides engineers and technicians in optimizing the design of new tractor products and supports the overall development process.

The HLA-based tractor virtual test system achieves the standardization of data format, technical framework, and model standards, resulting in enhanced interoperability and model reusability [17]. The system establishes a virtual test environment that accurately reflects real-life tractor operations. It enables virtual testing of various tractor tasks such as plowing, harrowing, and seeding. This system provides an effective solution to the challenges of extended testing cycles for new tractor products, pollution issues, and seasonal limitations, and it can yield positive economic outcomes.

### The architecture of the tractor virtual test system based on HLA

**Fig 1:**
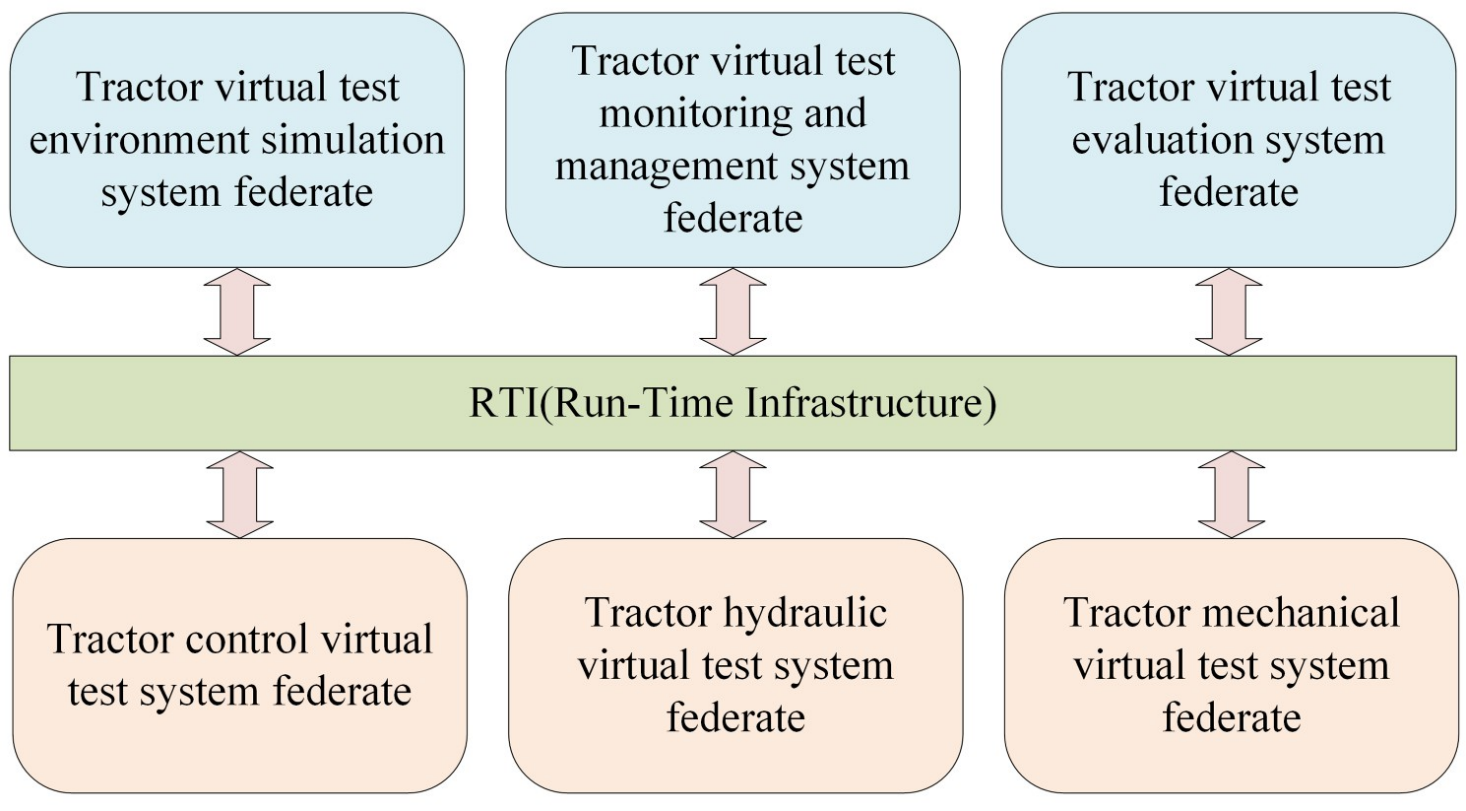
Structure diagram of the virtual test system for tractor based on HLA.

The architecture of the tractor virtual test system facilitates the communication between each functional subsystem of the tractor. It outlines the structural relationship between the virtual test system and the operational mechanism of the actual tractor system. Fig 1 visually represents the structure of the HLA-based tractor virtual test system.

## Tractor critical parts federate build

Federate play a crucial role within the federation, encompassing all the components involved in its execution. These members are responsible for integrating the subsystems of the simulation system into the federation as a whole, enabling their participation in the simulation. The tractor virtual test system is an integrated simulation system that combines multiple subsystems, virtual environment simulation, and virtual test monitoring and management. This comprehensive system involves numerous simulation objects, intricate models, and real-time data transmission. Consequently, it becomes imperative to address various challenges simultaneously, such as simulation time synchronization, human-machine interaction, model coordination, simulation monitoring and management, real-time analysis, and visualization of the simulation process, across all federate. A unified structure is required to effectively manage and coordinate interactions among the simulation systems [18]. Furthermore, each subsystem should possess a standardized architecture internally. Leveraging the reusability and interoperability advantages of the HLA can meet the specific demands of the tractor virtual test system while providing support for future system upgrades.

According to the HLA system design rules, the tractor virtual test system is a federation. It comprises various members, including the tractor control virtual test system, tractor hydraulic virtual test system, tractor mechanical virtual test system, tractor virtual test environment simulation system, tractor virtual test monitoring, and management system, tractor virtual test evaluation system, and other members within the federation. These members primarily participate in the virtual testing of key components related to tractor control, hydraulic systems, and mechanical aspects. These components encompass the tractor engine system, tractor transmission system, tractor driving system, tractor steering system, tractor brake system, and tractor power output system. Each member of the federation fulfills specific functions as outlined below.

1. Federate of tractor control virtual test system. Including the sum of the tractor’s key components control system, sending control instructions to the corresponding controlled objects through run-time infrastructure (RTI), and receiving real-time feedback information from the controlled objects. Realize closed-loop control.
2. Federate of tractor hydraulic virtual test system. Including the sum of tractor key components hydraulic system, through RTI to receive signals from the control system or mechanical system. Realize the tractor hydraulic system to control the mechanical system.
3. Federate of tractor mechanical virtual test system. Including the sum of tractor key parts mechanical system, through RTI to receive signals from the control system or hydraulic system and feedback signals to the control system or hydraulic system. Realize the specific implementation of the tractor mechanical system.
4. Federate of tractor virtual test environment simulation system. Including a working condition simulation module and 3D viewing window module, it provides working condition simulation for the tractor virtual test process. Realize the tractor rotary tillage, plowing, harrowing, and other working conditions of the live view and data simulation.
5. Federate of tractor virtual test monitoring management system. Realize the real-time monitoring and management of the tractor virtual test process.
6. Federate of tractor virtual test evaluation system. Realize the evaluation of the tractor virtual test process and results, analyze the reliability of the tractor virtual test results, and label the simulation non-normal data.

## Simulation system FOM/SOM table design and federation development

The object model template (OMT) serves as a standardized structural framework for the HLA object model, facilitating the understanding and depiction of data collaboration and interaction among federate [19]. The OMT plays a crucial role in expressing the potential capabilities of federate, aiding the design and application of general object model development tools. Within HLA OMT, two primary types of object models are defined. The first is the federation object model (FOM), which describes multiple interoperable federate. The second is the simulation object model (SOM), which describes the characteristics specific to individual federate. The FOM encompasses the characteristics, object classes, and object class attributes of interaction classes, along with the parameters necessary for information exchange among all participating federate in the simulation. It serves the purpose of establishing a standardized format for data exchange within the simulation. On the other hand, the SOM outlines the characteristics of interactive classes, interaction class attributes, object classes, and object class attributes that can be published or subscribed to by a federate [20]. The SOM reflects the participation capabilities of the federate during the simulation runtime. The process of developing the FOM/SOM involves abstracting and modeling the federation’s interaction data. Federate publishes the interaction classes and object classes required by other participants. They then determine the order of the needed interaction and object classes, facilitating information interaction and interoperability between federate. The following is an example of each federate in the tractor virtual test system to introduce the FOM/SOM and federate interface design.

### Member object class and its attribute table

The member object instances involved in the federation interactions are called member object classes, and their composition contains the full functionality of the federate interactions. Through the analysis of the tractor virtual test system, Table 1 shows some objects involved in each federate. According to the specific attributes among the various types of federate, extract the common attribute to design general base classes, control classes, mechanical classes, hydraulic classes, etc., to facilitate the inheritance of other classes.

**Table 1:** FOM object class structure simple table.

Level 1	Level 2	Level 3	Level 4
Control class	Transmission system control part	Powershift transmission control part	Reversing clutch control part
			Shift clutch control part
		…	…
	Power output system control part	Power output shaft control part	Power output shaft speed control
			Power output shaft torque control
		…	…
	…	…	…
Machinery class	Transmission system mechanical part	Power shift transmission mechanical part	Reversing clutch mechanical part
			Shift clutch mechanical part
		…	…
	Power output system mechanical part	Power output shaft mechanical part	Power output shaft speed size
			Power output shaft torque size
		…	…
	…	…	…
Hydraumatic class	Transmission system hydraulic part	Powershift transmission hydraulic part	Hydraulic part of reversing clutch
			Hydraulic part of shift clutch
		…	…
	Power output system hydraulic part	Power output shaft hydraulic part	Power output shaft drive pressure size
			…
		…	…
	…	…	…

The next level object class both inherits the properties of the previous level object and has its object properties. Table 2 shows the property table of the main object mechanical class of the FOM table.

**Table 2:** FOM main object mechanical class property table.

Object class	Property Identification	Variable identification	Type	Unit
Machinery class	displacement	distance	double	mm
	force	force	double	N
	torque	torque	double	N.M
	rotation speed	rev	double	r/min
	angular velocity	palstance	double	rad/s
	angular acceleration	angular acceleration	double	rad/s2
	velocity	speed	double	m/s
	…	…	…	…

### Member interaction class and its parameter list

Member interaction classes denote the instances that actively engage in federation interactions. Within the HLA-based tractor virtual test system, interaction classes play a crucial role in governing interoperability among federate. They constitute a significant component of the overall interaction process in the HLA system [21]. Interaction classes and object classes collaborate to facilitate the implementation of the interaction process within the system. The repertoire of interaction classes encompasses control, mechanical, and hydraulic interaction classes, with specific details provided in Table 3.

**Table 3:** FOM main interaction class property table.

Interaction Class	Property	Describe
Control class	c_Rpressure signal_1	Reversing clutch pressure signal
	c_Spressure signal_1	Shift clutch pressure signal
	…	…
Machinery class	m_force_1	Piston pressure
	m_distance_1	Piston displacement
	…	…
Hydraumatic class	h_press_1	Hydraulic pressure
	h_traffic_1	Hydraulic flow
	…	…

### Interface specification

The HLA RTI provides a series of services for simulation interconnection. It is the basis for the HLA simulation system to achieve distributed simulation scalability and hierarchical management control and is also the core of the realization of the HLA simulation system. RTI provides effective support for the integration and management of simulation system operating status information. Through the interface specification, the HLA-based tractor virtual test architecture can better realize the simulation management and simulation operation. The six types of services provided according to the HLA architecture interface specification make the interoperability between the members of the federations easier to achieve and more reasonable [22]. The HLA-based tractor virtual test system contains six services.

1. Federate management services.
2. Ownership management service.
3. Declaration management service.
4. Object management service.
5. Data distribution service.
6. Time management services.

### Federate program development

**Fig 2:**
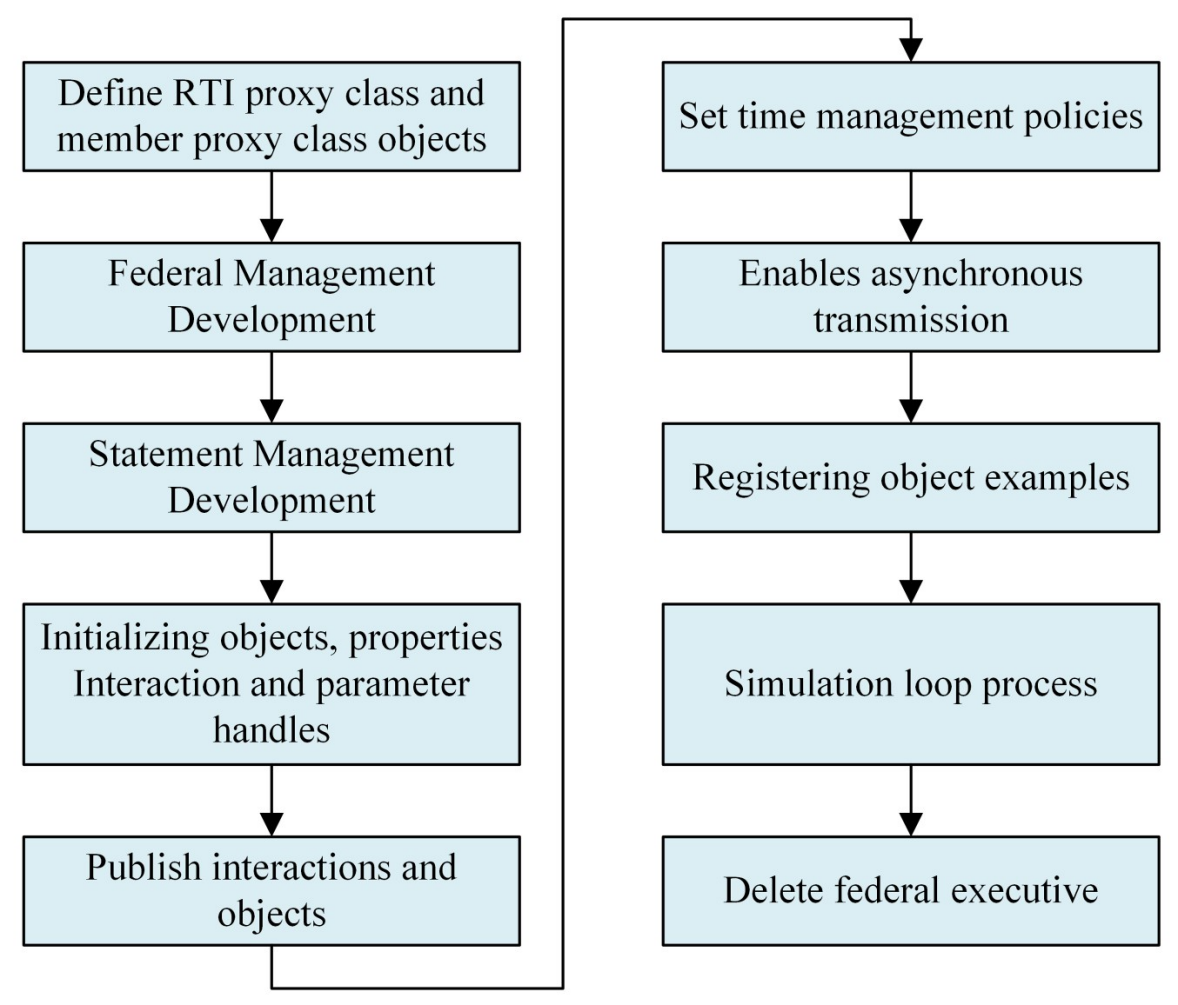
Federation program development process.

The selection of simulation software for the tractor virtual test environment, based on the HLA, proceeded as follows. The HLA’s RTI chosen for this endeavor is BH RTI, which stands for Beihang Run-Time Infrastructure. BH RTI represents an RTI software autonomously developed by the State Key Laboratory of Virtual Reality Technology and Systems at Beijing University of Aeronautics and Astronautics. In terms of federate, the control class opted for Simulink software, while the mechanical class embraced Adams software. For the hydraulics class federate, the choice fell upon AMEsim software. The software employed for program development is Visual Studio, with C++ serving as the program development language. A visual representation of the program development process for the federate can be observed in Fig 2.

The development of federation programs in BH RTI necessitates attention to including appropriate header files. Additionally, it is essential to place the federation execution data file (fed file) and the lrc dynamic link library file in the same directory as the federation application [23]. The development process for federation applications commences with defining the RTI agent class and member agent class objects, followed by the development of federation management. Federation management encompasses activities such as creating, revoking, joining, and exiting the federation. Claim management development primarily involves establishing the declaration information of the federate. Initialization objects, properties, interactions, and parameter handles are developed to provide technical support to the system. Publishing interaction and object development serves the purpose of configuring and publicly disseminating the interaction information of simulation systems. A time management strategy is implemented by defining each simulation subsystem to run the simulation based on a specific temporal logic. The development of registered object instances aims to enable various simulation subsystems to register objects through programming functions. The simulation loop process is developed to initiate the simulation loop within the simulation system. Finally, the function for deleting federated execution is implemented as part of the program development. Below is an illustrative code snippet for the federation program.

……

Virtual void creatFederationExecution // Create federation execution

(std::wstring const & Tractor virtual test system, // Federation executive name: Tractor virtual test system

std:: wstring const & Tractor virtual test systemFED) // Link to tractor virtual test system fed file)

……

virtual FederateHandle joinFederationExecution // Join federation executive

(std::wstring const & federateType,

std::wstring const & Tractor virtual test system,

Federate Ambassador & federate Ambassador,

LogicalTimeFactory & logicalTimeFactory,

LogicalTimeIntervalFactory & logicalTimelntervalFactory)

……

virtual void resignFederationExecution // Withdrawal from federation execution

……

### Interoperability among federate

**Fig 3:**
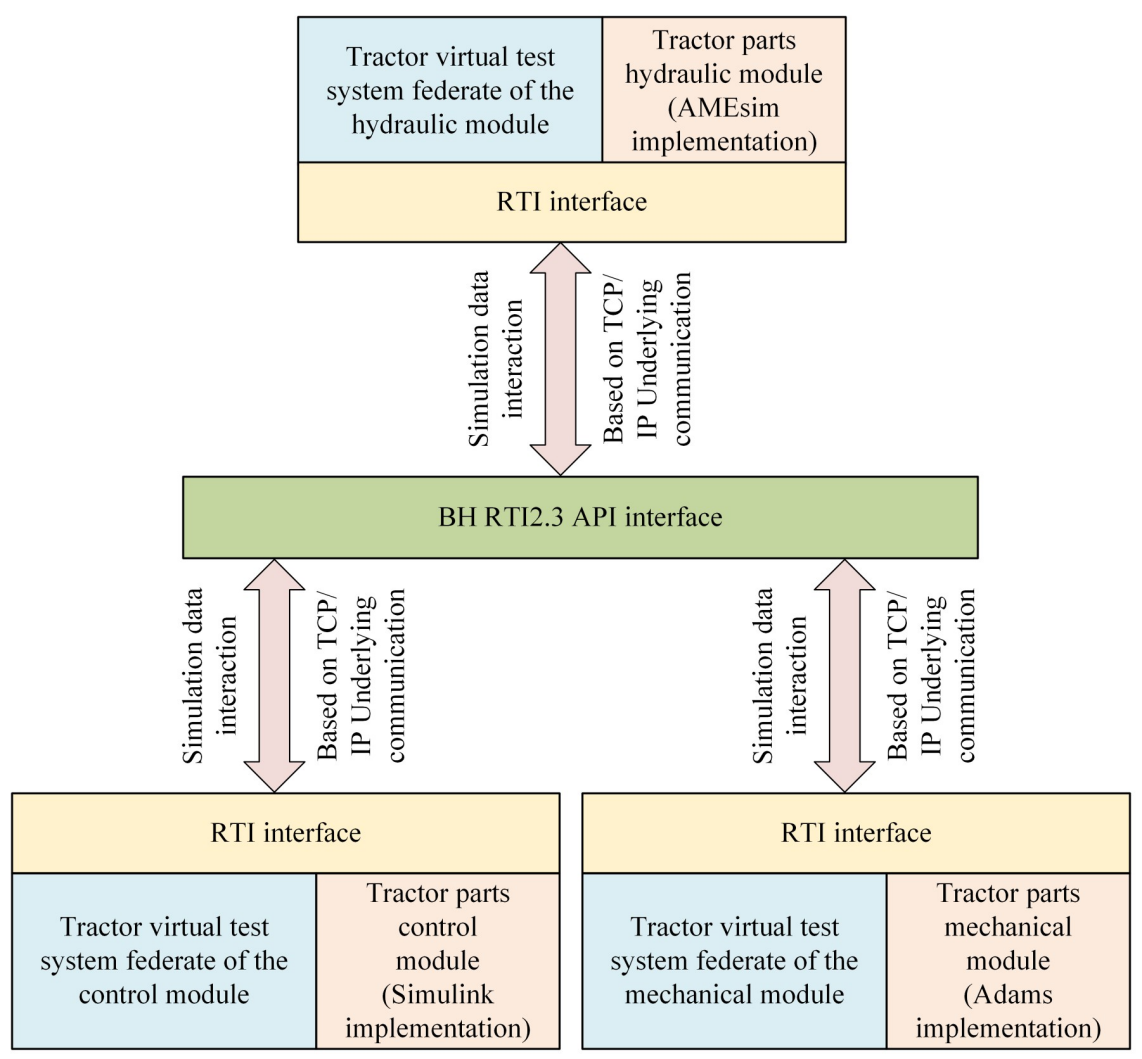
Schematic diagram of simulation data transmission.

The federation of the tractor virtual test system engages in information exchange facilitated by TCP/IP-based communication. Fig 3 illustrates the transfer of simulation data across the federation. The simulation process is initiated by executing the Simulink control model, responsible for distributing hydraulic system control signals to the BH RTI using the RTI interface. The AMEsim hydraulic model subscribes to these control signals via the RTI interface and subsequently publishes the corresponding hydraulic signals back to the BH RTI. In parallel, the Adams mechanical model subscribes to the hydraulic signals transmitted through the RTI interface from the BH RTI, facilitating the execution of the dynamic simulation. Following the simulation, the results are accessible and can be examined through the dedicated simulation software.

**Fig 4:**
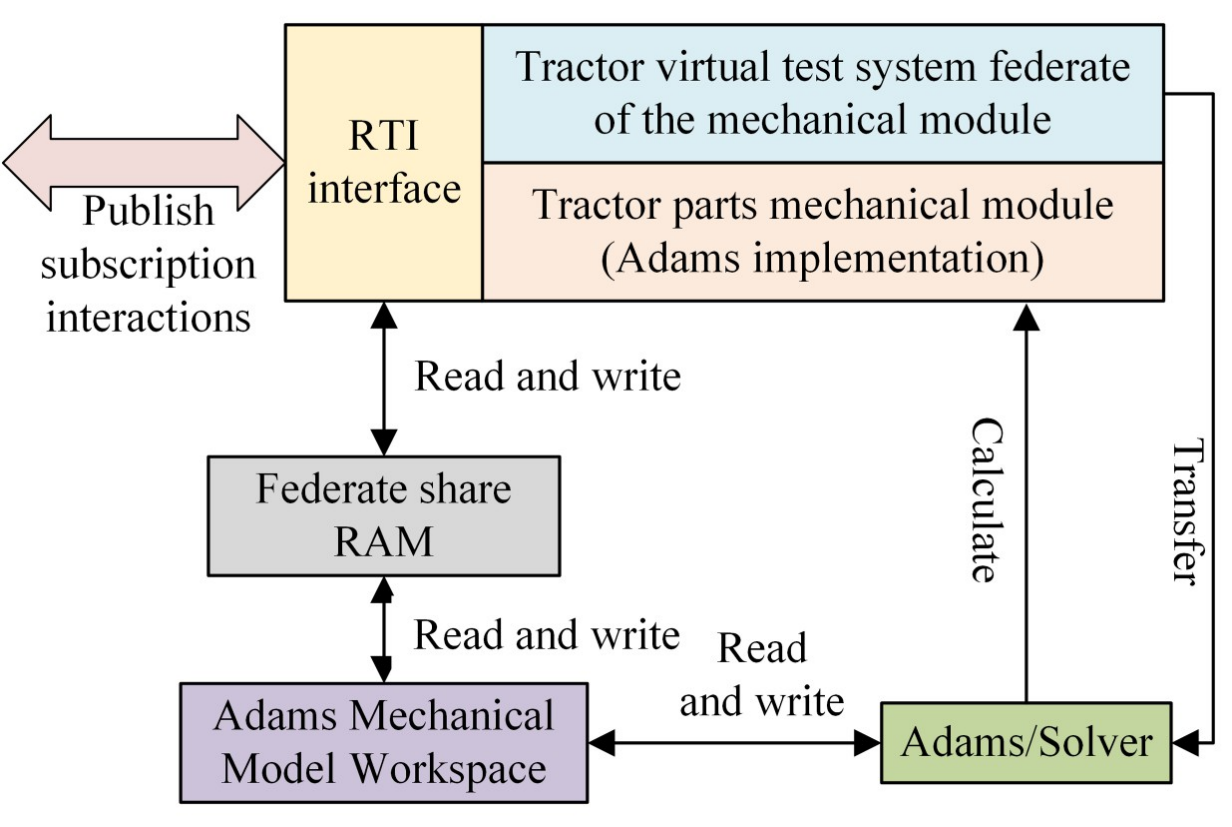
Schematic diagram of the tractor virtual test system mechanical module federate federation execution.

Fig 4 shows the schematic diagram of the mechanical module federate of the tractor virtual test system during the federation execution. The control module and hydraulic module federate are performing distributed simulations similar to the mechanical module federate, except that they also need to invoke Simulink and AMEsim interface commands and use their respective solvers to perform the solution. The operation of the simulation module by the federate mainly involves invoking the solver and receiving, storing, and sending simulation data. The federate of the mechanical module of the virtual test system starts Adams and solves the mechanical dynamics model by subscribing to the hydraulic signals from AMEsim via the BH RTI (written to the shared memory of the Federate via the RTI interface function). The solved data is written to the Adams mechanical model workspace and then to the federate’ shared memory space to complete the distribution and subscription of information [24].

**Fig 5:**
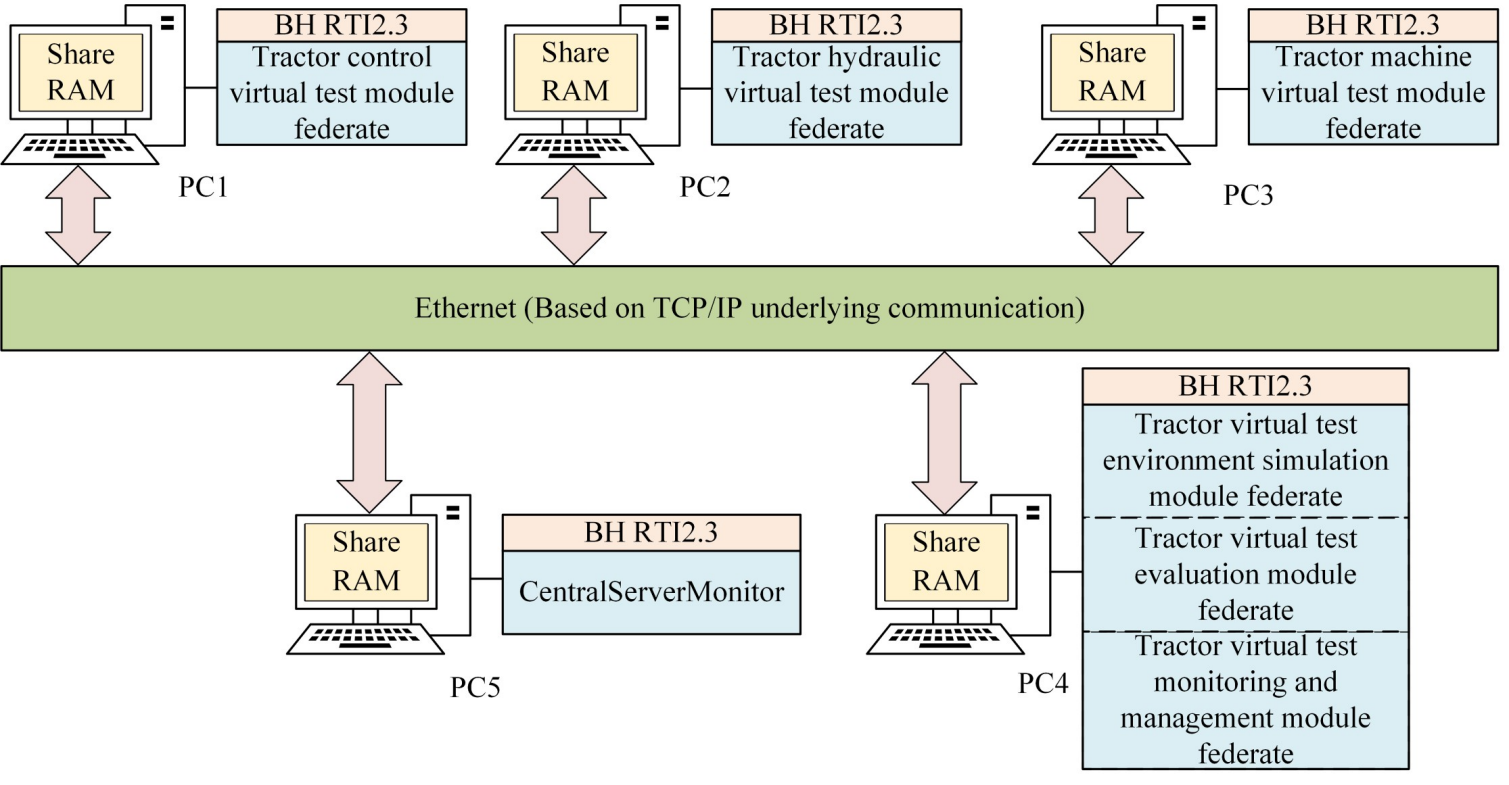
Tractor virtual test system hardware platform.

The structure of the virtual test system based on HLA, established the hardware platform of the tractor virtual test system, as shown in Fig 5.

In Fig 5, the computer performance parameters are 64-bit Windows 10 operating system, running memory 8G, 2.5GHz CPU frequency. Five computers are connected via Ethernet.

PC1 running HLA RTI software BH RTI, tractor control module model. PC2 running HLA RTI software BH RTI, tractor hydraulic module model. PC3 running HLA RTI software BH RTI, tractor mechanical module model.

PC4 running HLA RTI software BH RTI, tractor virtual test environment simulation module federate, tractor virtual test monitoring and management module federate, tractor virtual test evaluation module federate, data storage between components using shared memory.

The PC5 runs the HLA RTI software BH RTI and the BH RTI central server CentralServerMonitor.

### Tractor virtual test system operation process

The operation of the tractor virtual test simulation system is managed and controlled by the simulation master member. When the simulation starts, the BH RTI central server CentralServerMonitor is started first, and then the BH RTI core program of each federate computer is started. After that, the tractor virtual test simulation federation is created through the federation management application, and each federate joins the federation, and then the simulation cycle starts. At the end of the simulation cycle, each federate exits the federation and then destroys the federate, and the tractor virtual test simulation ends.

## Tractor virtual test system performance test

**Fig 6:**
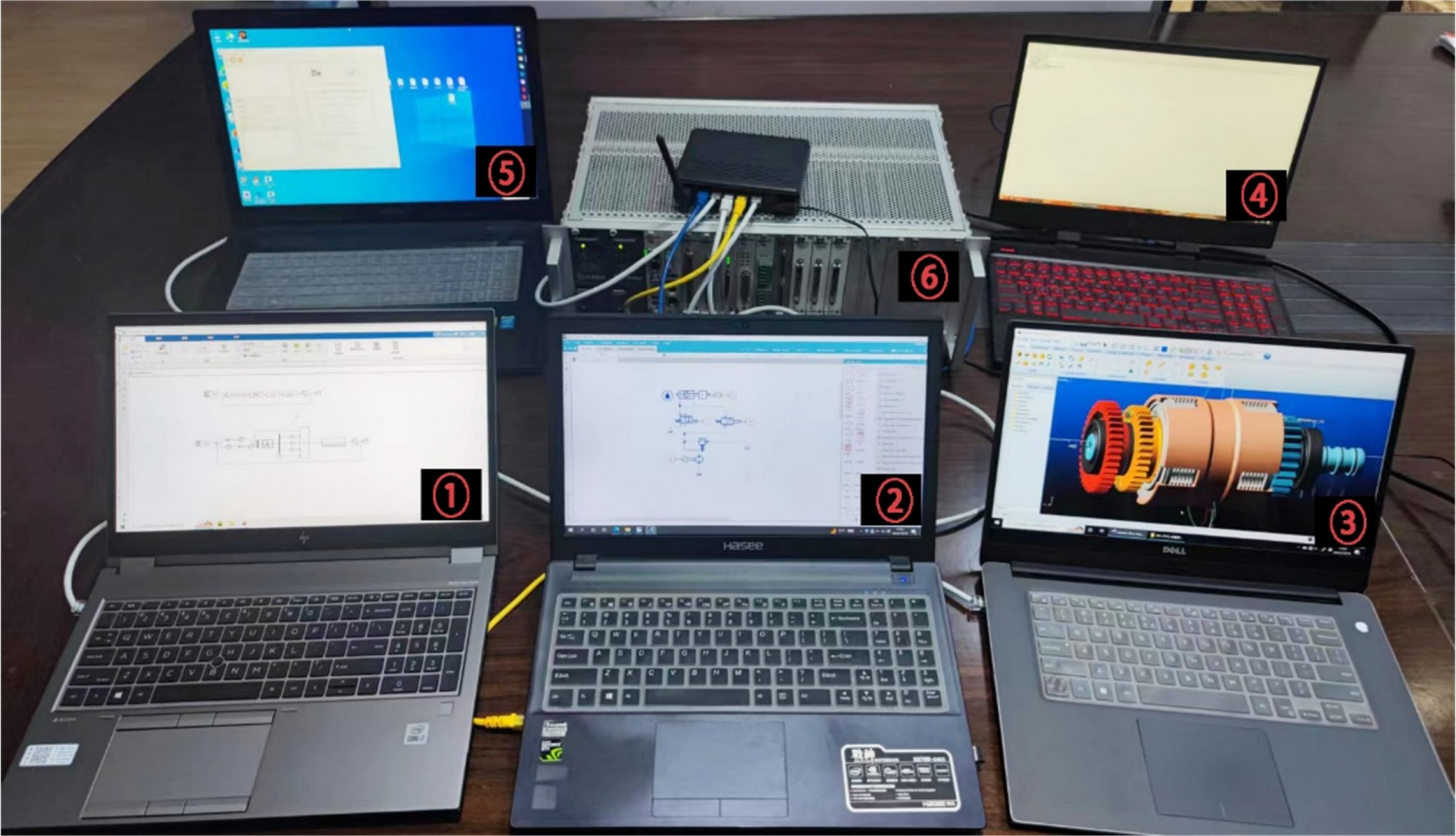
Tractor virtual test system. 1-PC1; 2-PC2; 3-PC3; 4-PC4; 5-PC5; 6-Data acquisition con-troller.

**Fig 7:**
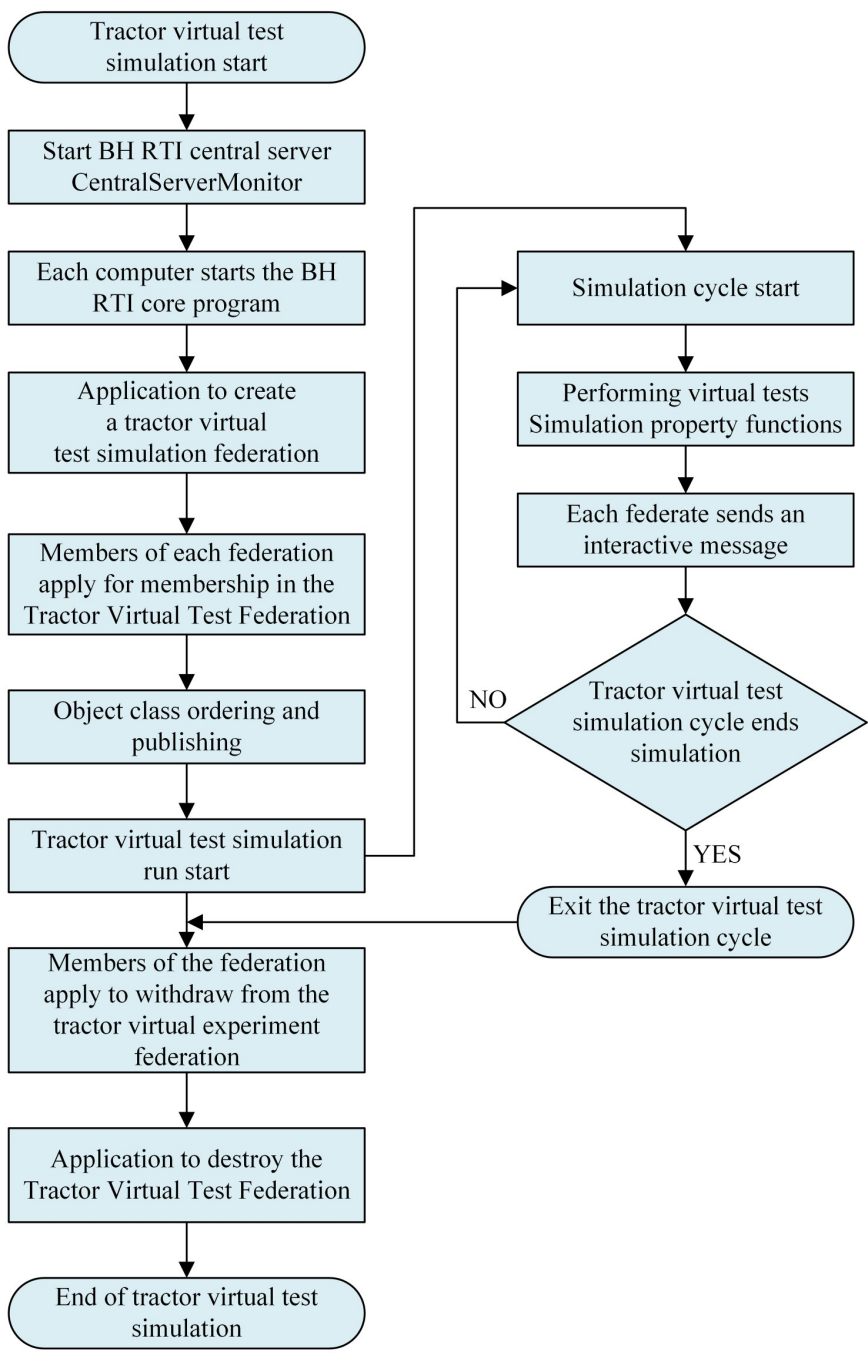
The operation process of the tractor virtual test system.

In Fig 6, the configuration and running programs of the five distributed computers PC1, PC2, PC3, PC4, and PC5 remain the same as in Fig 7. PC1 runs the BH RTI software and the model of the reversing clutch control system, PC2 runs the BH RTI software and the model of the hydraulic module of the reversing clutch, and PC3 runs the BH RTI software and the model of the mechanical module of the reversing clutch [25]. PC4 runs BH RTI software, tractor virtual test environment simulation module federate, tractor virtual test monitoring and management module federate, and tractor virtual test evaluation module federate. PC5 runs BH RTI software and BH RTI central server CentralServerMonitor. The data acquisition controller supervisor software Links-RT runs as a federate in PC4. The data acquisition controller host computer software Links-RT runs in PC4 as a federate.

### Virtual test of tractor PST reversing clutch

**Fig 8:**
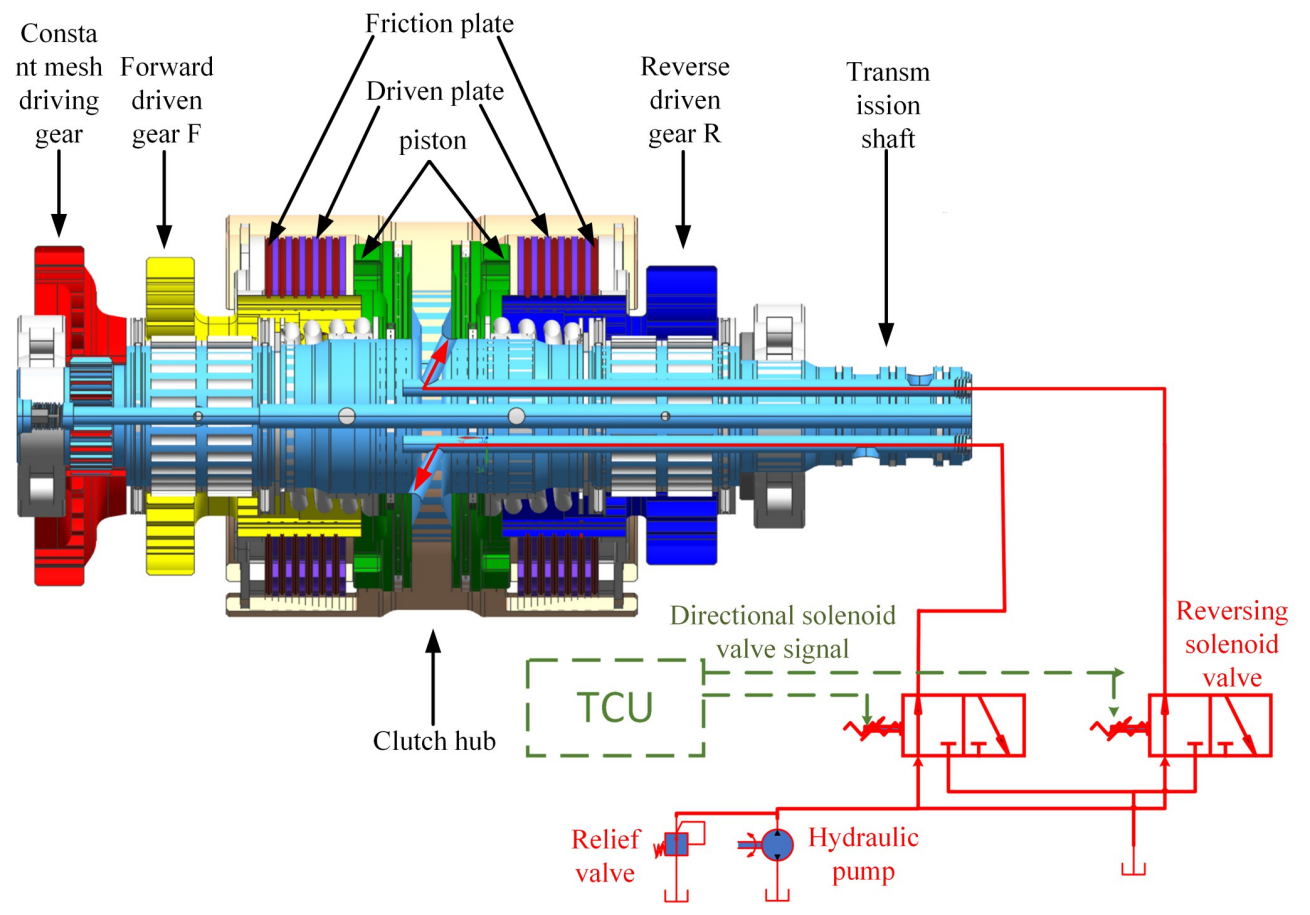
Schematic diagram of reversing clutch and power shuttle.

Tractor power shuttle refers to the uninterrupted power transmission during the forward and reverse gear switching process of the tractor. It is a necessary technology for the tractor to achieve high-quality and efficient frequent round-trip work and typical working conditions. As shown in Fig 8, the tractor power shuttle is achieved by separating and combining the forward-driven gear F and reverse-driven gear R of the reversing clutch with the clutch-driven plate. When the tractor is moving forward, the piston on the F side of the forward-driven gear is filled with oil. Combine the friction plate connected to the forward-driven gear F with the driven plate connected to the clutch hub. The power is transmitted to the forward-driven gear F through the transmission shaft, clutch hub, driven plate, and friction plate through the long mesh driving gear. When the tractor driver switches from a forward gear to a reverse gear, the TCU sends a power shuttle control signal to change the position of the solenoid valve. At this point, the piston on the F side of the forward-driven gear begins to return oil, and under the action of the return spring, the driven plate and friction plate on the F side of the forward-driven gear separate. At the same time, the piston on the R side of the reverse-driven gear begins to charge oil. The piston compresses the return spring, sliding and grinding the friction plate connected to the reverse gear driven gear R, and the driven plate connected to the clutch hub until fully engaged. The power is switched from the forward-driven gear F to the reverse-driven gear R, and the power shuttle is now complete.

**Fig 9:**
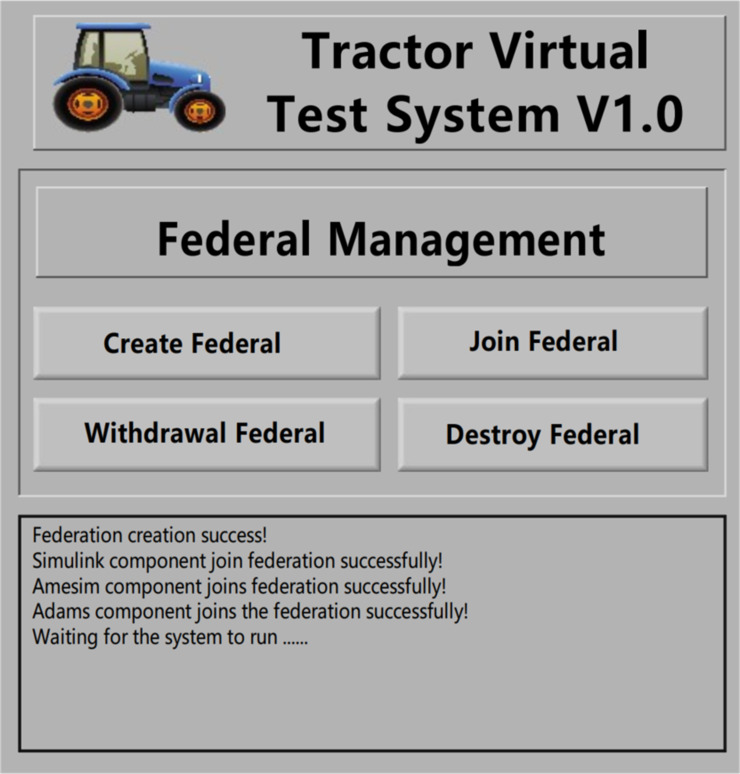
Reversing clutch virtual test interface.

**Fig 10:**
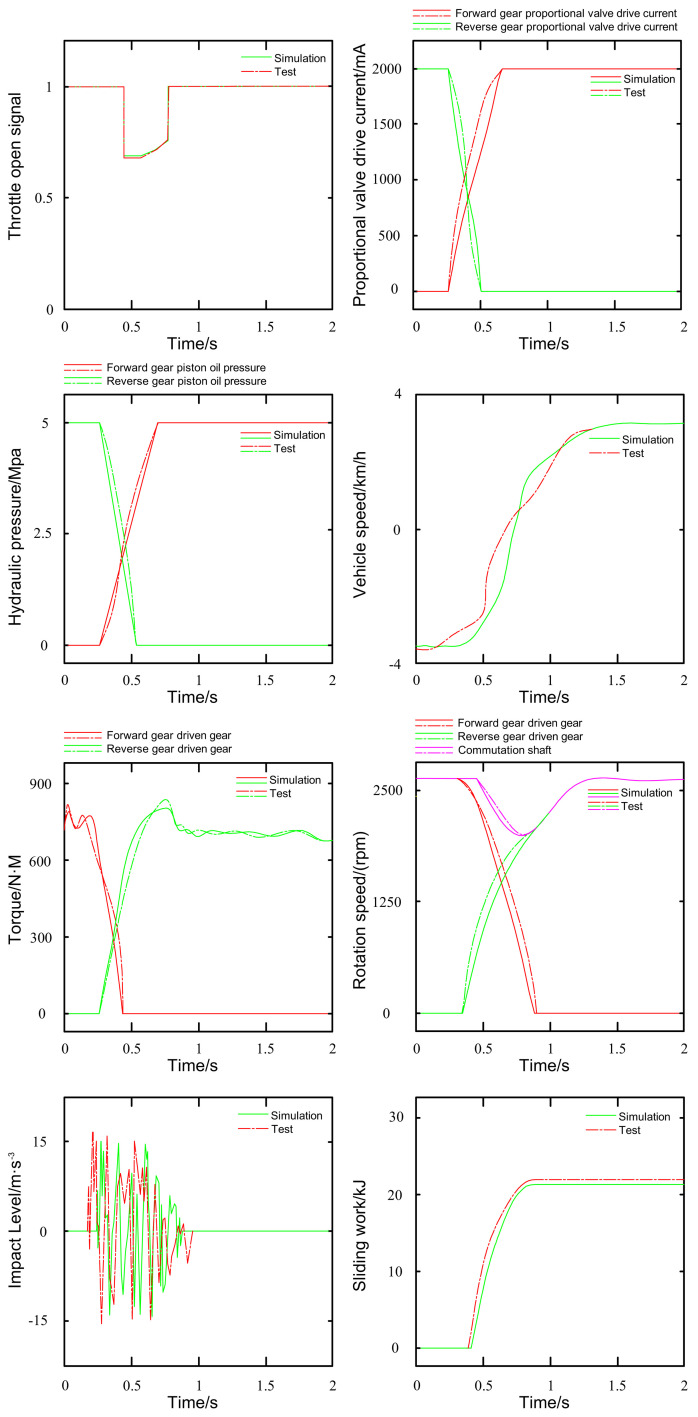
Simulation results of the 1st gear power reversing process.

The PST transmission system adopts the TX4A transmission system of the DongFangHong LF2204 tractor, and the reversing clutch model is ZCH95. The virtual test of the tractor PST reversing clutch is carried out through the HLA-based tractor virtual test system. The PST reversing clutch used for verification has undergone actual tests, and the test results meet the design standards of the PST reversing clutch. The interface of the reversing clutch virtual test system is shown in Fig 9, and each computer running program is configured according to Fig 6. The clutch condition selects the 1st gear heavy-load full throttle reverse gear shift as the forward gear, and the simulation results are shown in Fig 10.

The simulation and data transfer processes are outlined as follows. Once the tractor control unit (TCU) receives the signal to shift from forward gear to reverse gear, the Simulink-based module for controlling the tractor reversing clutch triggers the simulation process. Subsequently, the hydraulic signal is transmitted through BH RTI (Bus and Hub Real-Time Interface) to the hydraulic simulation module built-in AMEsim, initiating the hydraulic simulation of the reversing clutch [26]. Through BH RTI, AMEsim transfers the pressure signal to the reversing clutch dynamics simulation module developed in Adams, enabling the execution of the dynamic simulation. This comprehensive virtual testing procedure of the tractor’s PST reversing clutch yields the primary simulation data pertinent to its performance.

The simulation results are depicted in Fig 10, where the engine output torque undergoes a transition between the two clutches. The combined clutch associated with the reverse gear disengages from the commutation shaft, while the clutch engaged with the forward gear connects with the commutation shaft, affecting the ultimate power transfer [27]. This power reversal process spans 0.7 seconds, during which the speed shifts from -3.5 km/h to 3.5 km/h. The oil pressure in the forward gear piston increases from 0 MPa to 5 MPa, resulting in a peak impact acceleration of 17 m/s^3, with a total slippage work of 21 kJ incurred throughout the reversal process. It’s worth noting that the entire power reversal process exhibits a relatively smooth performance. To analyze the time series data generated from the HLA-based tractor virtual test and the physical test, statistical characterization was conducted using the grey-scale correlation method. This analysis yielded various statistical parameters, including the maximum value, minimum value, mean value, standard deviation, and root mean square value, for the two data sets. These parameters were utilized to construct two series, denoted X_i_ and Y_i_, expressed as follows.


$$X_{i}=[x_{1},\;x_{2},\;x_{3},\;x_{4},\;x_{5}]$$ (1)

$$Y_{i}=[y_{1},\;y_{2},\;y_{3},\;y_{4},\;y_{5}]$$ (2)

X_i_ and Y_i_ correlation degree is calculated as.


$$z_{i}=\frac{\min|x_{i}-y_{i}|+\xi \max|x_{i}-y_{i}|}{\left|x_{i}-y_{i}|+\xi \max|x_{i}-y_{i}\right|}$$ (3)

In the formula, z_i_ is the correlation coefficient of each feature parameter. ξ is the resolution coefficient and takes the value of 0.5 [28].

The correlation coefficient z of the virtual and physical test data is the average of the correlation coefficients of all characteristic parameters and is calculated as.


$$z=\frac{1}{n}\sum_{i=1}^{n}z_{i}$$ (4)

For z ∈ (0.9, 1], the virtual and physical test data are highly correlated. z ∈ (0.7, 0.9], the virtual and physical test data are more correlated. z ∈ (0.5, 0.7], the virtual and physical test data are highly correlated. z ∈ (0.3, 0.5], the virtual and physical test data are less correlated. z ∈ [0, 0.3], the virtual and physical test data are less correlated. z ∈ [0, 0.3], the virtual and physical test data are less correlated. z ∈ [0, 0.3], the correlation between the virtual test data and the physical test data is small.

After calculation, the average correlation coefficient z = 0.92. The virtual test data and the physical test data correlate well. The virtual test results are consistent with the bench test results. Through the virtual test and bench test comparison results analysis, the tractor PST reversing clutch virtual test model on the HLA-based tractor virtual test system works well.

### Performance test of data transmission delay of tractor virtual test system

The data transmission delay parameter reflects the system’s ability in real-time and data transmission capacity and is an important index parameter of the tractor virtual test system. According to the virtual test advance step and hardware device response time, the system time delay should not exceed 10ms.

To assess the influence of varying numbers of object attributes on system data latency performance, we established a 60-node tractor virtual test system. Each node represents a data size of 100KB, effectively emulating 60 attributes within the simulation system. The methodology employed for conducting these tests is outlined as follows.

1. Start the tractor virtual test system and complete the system initialization.
2. Simulink, AMEsim, and Adams each run data transfer, from Simulink software output 1 control signal to AMEsim software, and then by AMEsim software to Adams software, Adams software to Links-RT, start timer recorded as time T1 moment.
3. Links-RT receives the signal and feeds back the signal to the federate Simulink.
4. Repeat steps (2) and (3) 100 times, stop the timer, and count as time T2.
5. Test to obtain the time delay under the data size corresponding to the 1st control signal as ((T2-T1)/200)s.
6. Repeat steps (2) ~ (5), and increase the transmission line in turn to obtain the time delay under the data size corresponding to the 2-way and 3-way……60 signals.

**Fig 11:**
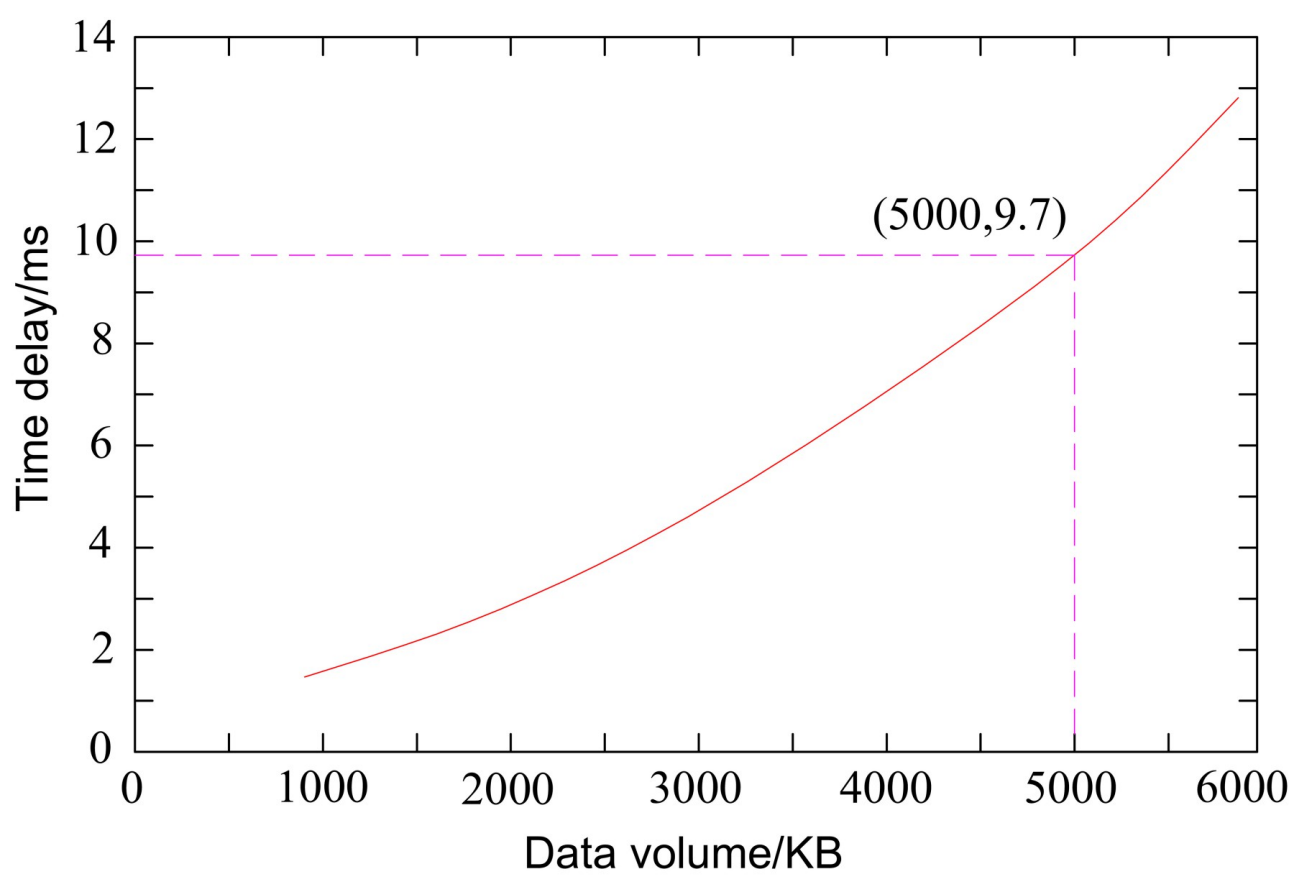
Data transfer delay results.

Fig 11 presents the results obtained from the test regarding data transmission delays.

In Fig 11, when the system transmission data volume reaches 5000KB, the time delay is 9.7ms. It meets the real-time performance requirement that the maximum delay of the tractor virtual test system does not exceed 10ms, and the data transmission delay performance is acceptable [29].

## Conclusion

This paper presents a detailed study and design of a distributed virtual test system for tractors based on HLA. The problems of low tractor test efficiency and long new product development cycle are solved, and the main results are as follows.

1. Analyzing logic and hardware structure of the tractor virtual test system. Design federation FOM and SOM table of the tractor virtual test system. Study interoperability among the federate and the system operation flow in detail.
2. The performance of the HLA-based tractor virtual test system is stable. When the system data volume reaches 5000KB, the data delay is 9.7ms, which meets the required tractor virtual test delay of no more than 10ms.
3. The HLA-based tractor virtual test system makes efficient use of the existing tractor model and has certain advantages in model reuse, interoperability, and system expansion.
